# Influence of Draft Angle Design on Surface Texture–Dimensional Accuracy Coupling in Injection-Molded Commodity and Engineering Polymers with Semi-Crystalline and Amorphous Characteristics

**DOI:** 10.3390/polym17212892

**Published:** 2025-10-29

**Authors:** Hui-Li Chen, Po-Wei Huang, Yu-Shan Huang

**Affiliations:** 1 Mechanical Engineering, College of Semiconductor Engineering, CTBC University of Technology, Tainan 74448, Taiwan; 2 Mechanical and Automation Engineering, College of Engineering, Taiwan Steel University of Science and Technology, Kaohsiung 821013, Taiwan; bowei8915@gmail.com; 3 Plastic Precision Machining Center, College of Semiconductor Engineering, CTBC University of Technology, Tainan 74448, Taiwan

**Keywords:** plastics injection molding, crystalline and amorphous polymers, draft angle design, surface texture replication, dimensional accuracy

## Abstract

In injection molding, draft angle design plays a critical role in ensuring smooth de-molding and maintaining surface quality. With the growing emphasis on aesthetics and the increasing demand for the appearance of plastic products, the need for textured plastic components has continuously risen. The coupling between surface texture replication and dimensional accuracy has become an important indicator of product performance. However, systematic studies on the interaction between different polymer materials and draft angle design remain limited. This study aims to investigate the influence of draft angle variation on the surface texture quality and dimensional stability of injection-molded parts by comparing the differences between crystalline and amorphous thermoplastic materials, as well as between commodity and engineering plastics. Four representative polymers, namely polypropylene (PP), polyoxymethylene (POM), acrylonitrile-butadiene-styrene (ABS), and polycarbonate (PC), were selected to examine the impact of material characteristics on surface texture replication after molding. In addition, product geometries incorporating eight draft angles (0° to 3.5°) were designed. Surface texture replication was analyzed using scanning electron microscopy (SEM) and surface profilometry, while dimensional deformation was measured with a high-precision optical measuring instrument. The results show that draft angle variation has a limited influence on the overall trend of dimensional deformation, but it has a significant effect on the clarity of surface replication. Crystalline polymers exhibited generally higher surface roughness than amorphous polymers, and the distinction between commodity and engineering plastics, particularly those requiring higher processing temperatures, also led to higher roughness (PP > POM; ABS > PC). Dimensional deformation was more pronounced in crystalline polymers (POM > PP > ABS > PC). SEM observations further confirmed that higher roughness corresponded to clearer and more distinguishable texture patterns, whereas lower roughness resulted in blurred or indistinct textures.

## 1. Introduction

**Figure 1:**
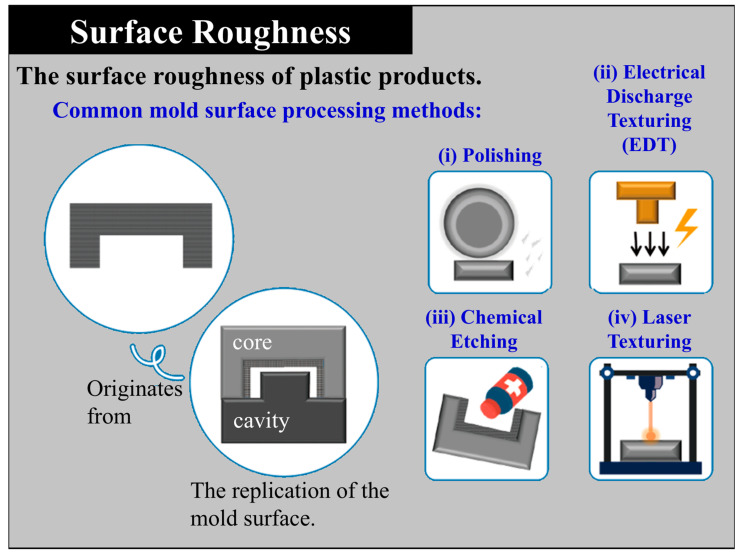
Schematic of mold surface of the replication and common processing methods.

With the continuous advancement of global industrial technologies and the rapid evolution of consumer markets, plastic products have become indispensable in diverse fields such as automotive, electronics, household appliances, medical devices, sports equipment, and daily commodities. Plastics have gradually replaced traditional materials such as metals and wood [1,2,3], establishing themselves as critical materials supporting modern industry and daily life. Injection molding, owing to its high production efficiency, high level of automation, ability to manufacture complex geometries, and low unit cost, has emerged as the most representative processing technology within the plastics industry. As consumer demands have shifted from functionality alone to a balance of performance, aesthetics, and quality, both designers and manufacturers are increasingly required to ensure stable surface quality and refined product appearance. In high-value-added sectors such as consumer electronics housing, automotive interiors, and household appliances, the external appearance of a product is no longer merely an extension of its structural integrity but has instead become a symbol of brand image, user experience, and market competitiveness. For end-users, factors such as whether a product surface presents uniform gloss, fine textures, and freedom from scratches or deformation caused during demolding directly affect purchase decisions (Figure 1).

With the rise of aesthetic-driven trends, surface treatment has evolved from basic glossy or matte finishes to more diversified and differentiated surface textures [4,5,6,7,8], allowing injection-molded parts to satisfy both functional and aesthetic requirements. Consequently, achieving stable appearance quality while maintaining manufacturing efficiency has become a focal issue in both academic research and industrial applications in recent years. Among various surface treatment technologies, mold surface texturing has become the most widely adopted and cost-effective approach. Texture is created on the mold cavity surface through processes such as etching, sandblasting, or chemical treatment, enabling the replication of specific geometric or leather-like patterns on molded plastic surfaces. This technique not only enhances the visual and tactile qualities of products but also effectively conceals molding defects such as weld lines, sink marks, and flow marks [9], thereby improving yield and market value. Furthermore, surface textures serve functional purposes by enhancing wear resistance, scratch resistance, anti-glare performance, and grip comfort, which explains their widespread use in automotive components, 3C electronic housings, and medical devices where surface quality is critical. However, texture replication is not determined solely by the precision of mold fabrication. Its success is strongly influenced by multiple factors within the injection molding process, including processing parameters, mold design, material properties, and demolding conditions [10,11]. Among these, draft angle design is a crucial determinant of whether products can be smoothly demolded and whether surface textures can be faithfully replicated [12,13,14,15]. Insufficient draft angles often lead to scratches or damage to the textured surface during demolding, or even deformation caused by excessive ejection force. Conversely, overly large draft angles may compromise dimensional accuracy and conflict with design requirements. Thus, determining appropriate draft angles tailored to specific material characteristics and surface appearance requirements has become a central factor affecting the quality of textured surfaces.

Due to the inherent differences in molecular structures and physical properties, polymer materials exhibit distinct cooling shrinkage behaviors, surface flowability, and interfacial friction with mold walls during injection molding. Semi-crystalline polymers such as polypropylene (PP) and polyoxymethylene (POM) possess ordered molecular chain arrangements during cooling, leading to higher shrinkage rates, greater susceptibility to dimensional deformation and warpage, but also more pronounced surface roughness and clearer texture replication. In contrast, amorphous polymers such as acrylonitrile butadiene styrene (ABS) and polycarbonate (PC) exhibit disordered molecular arrangements, resulting in lower shrinkage, better dimensional stability, but reduced clarity in texture replication. From an application perspective, commodity plastics are widely used due to their low cost and ease of processing, but they typically have limited thermal stability and mechanical strength. Engineering plastics, by comparison, provide superior heat resistance, dimensional precision, and mechanical performance, making them suitable for high-performance applications. Despite these distinctions, systematic experimental data and design guidelines regarding the combined effects of draft angle design and polymer classification on texture replication and dimensional accuracy remain insufficient. Addressing this gap, this study investigates the influence of draft angle variation on the coupling between surface texture and dimensional stability across different polymer categories. Representative materials, namely PP, POM, PC, and ABS, were selected, and multiple geometries with varying draft angles were designed. Surface replication was evaluated through scanning electron microscopy (SEM) and surface profilometry, while dimensional deformation was assessed using high-precision optical measurement. By systematically analyzing the effects of draft angle and material type on surface texture clarity and dimensional accuracy, this research establishes practical design guidelines for textured mold development and provides theoretical contributions for further exploration of draft angle-material interactions. Ultimately, the findings aim to assist both industry and academia in optimizing injection-molded surface aesthetics while maintaining dimensional precision.

## 2. Materials and Methods

### 2.1. Geometrical Product and Its Mold Design

**Figure 2:**
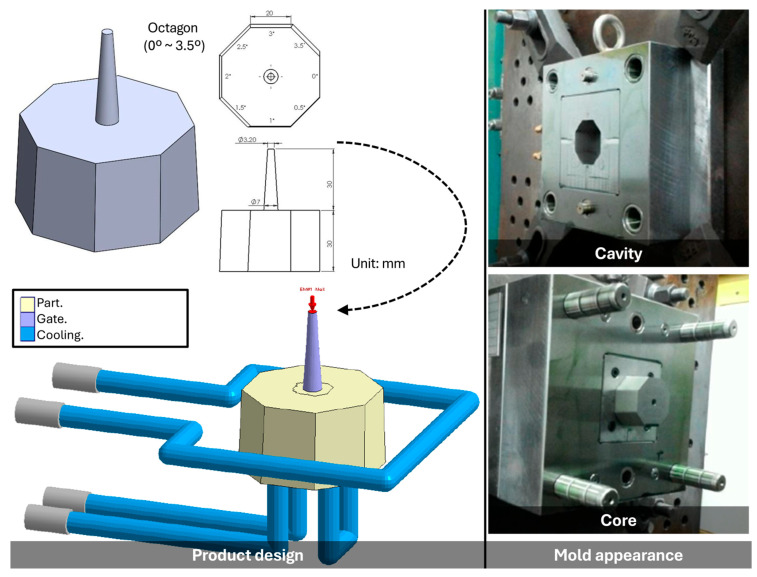
Specimen design with different draft angles (including gates and cooling channels) and mold.

To investigate the effects of draft angle on surface quality and dimensional accuracy, specimens of PP, POM, ABS, and PC were molded using a specially designed tool incorporating multiple draft angle geometries (Figure 2). The specimen was configured as an octagonal prism with an overall height of 30 mm (excluding the sprue).

Starting at 0°, each subsequent side increased by 0.5° in a clockwise sequence, resulting in eight distinct draft angles. Each side of the octagon had a width of 20 mm. The octagonal geometry was chosen because it allows eight draft angles to be integrated within a single specimen, thereby eliminating variations caused by different molds or molding cycles. This design not only ensures consistent processing conditions across all draft angles but also enables direct comparison of surface texture replication and dimensional accuracy under controlled, repeatable conditions. The specimen cavity surface was treated with Electrical Discharge Texturing (EDT) (Figure 1ii), applied on the core (mother mold) side. EDT provides uniform roughness, stable quality, and cost-effective large-area texturing. Compared with polishing, chemical etching, and laser texturing, it offers superior reproducibility and is best suited for matter, anti-glare, and tactile plastic products.

### 2.2. Molding Material and Its Equipment

**Figure 3:**
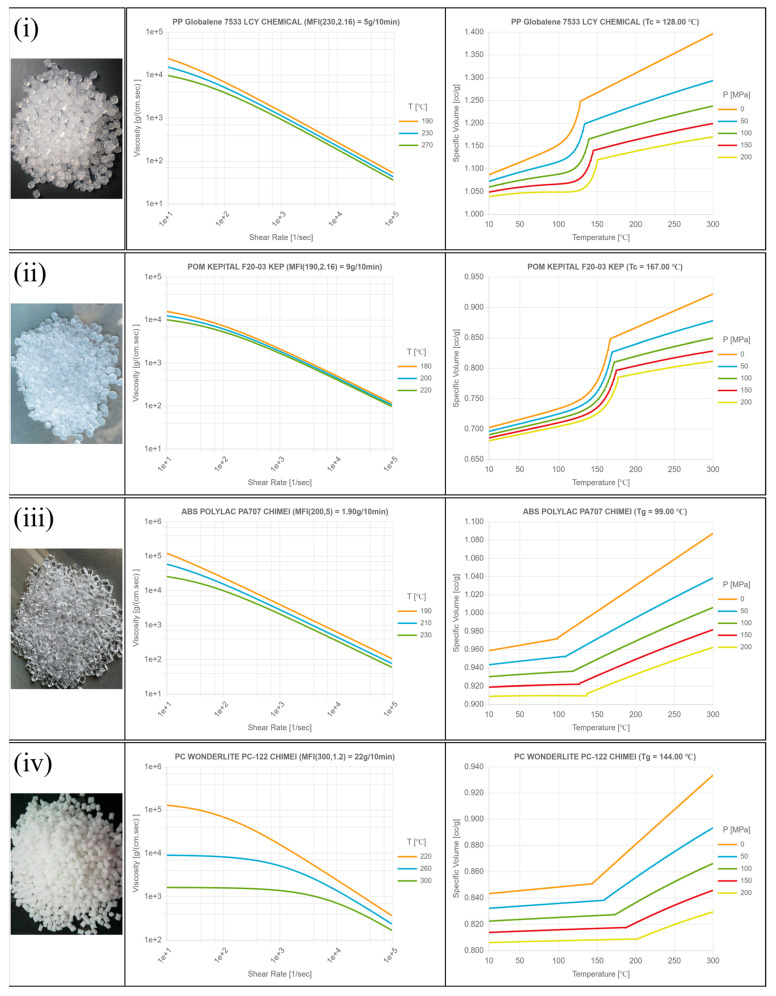
Schematic of viscosity and P-V-T relation of experiment materials: (**i**) polypropylene (PP); (**ii**) polyoxymethylene (POM); (**iii**) acrylonitrile-butadiene-styrene (ABS); (**iv**) polycarbonate (PC). (Source: Moldex3D Information Library).

The molding materials employed in this study comprised four representative thermoplastics encompassing both semi-crystalline and amorphous categories (Figure 3). Specifically, polypropylene (PP-6331, LCY Group, Taipei, Taiwan; semi-crystalline) with a melt flow index (MFI) of 5 ± 0.5 g/10 min, polyoxymethylene (POM-F2003, Mitsubishi Engineering-Plastic Corporation, Tokyo, Japan; semi-crystalline) with an MFI of 9 ± 0.5 g/10 min, acrylonitrile-butadiene-styrene (ABS-PA707, CHIMEI Industry Co., Ltd., Tainan, Taiwan; amorphous) with an MFI of 1.9 ± 0.5 g/10 min, and polycarbonate (PC-122, CHIMEI Industry Co., Ltd., Tainan, Taiwan; amorphous) with an MFI of 22 ± 0.5 g/10 min were selected. These materials are widely used in industrial practice and were chosen to represent the fundamental differences between semi-crystalline and amorphous polymers in terms of molecular arrangement, cooling shrinkage, and flow behavior. On the other hand, it can be seen that the viscosity variety and pressure–volume–temperature (P–V–T) characteristics of the materials are different due to the relationship of crystallinity (Ref: Moldex3D information library). In particular, PP and POM exhibit ordered molecular packing during cooling, which results in higher shrinkage and rougher surfaces, while ABS and PC, as amorphous polymers, display disordered molecular chains associated with lower shrinkage but reduced texture replication clarity. Prior to molding, all pellets were dried for 4 h at 80 °C to prevent moisture-induced degradation. From a theoretical perspective, the distinct behaviors of semi-crystalline and amorphous polymers can be attributed to their crystallization mechanisms and thermal transitions. Semi-crystalline polymers undergo spherulitic crystallization during cooling, which leads to heterogeneous shrinkage and localized residual stress. These phenomena often manifest as increased surface roughness and greater dimensional inaccuracy, particularly when cooling is non-uniform or draft angles are insufficient to reduce demolding friction. In contrast, amorphous polymers solidify through the glass transition without long-range molecular ordering, producing lower volumetric shrinkage and enhanced dimensional stability. However, the absence of crystalline lamellae reduces the material’s ability to fully replicate fine surface textures, resulting in less distinct or blurred surface patterns. In addition, the injection machine (basic model CLF-60TX) used in this study was jointly developed by the researchers and CLF (Chuan-Lih-Fa Machinery Works Co., Ltd.; Tainan City, Guanmiao District, Taiwan). This system incorporated an accurate injection controller and real-time monitoring module to ensure process stability, plasticizing quality, and precise screw position control. The machine provided a maximum injection rate of 115 cm^3^/s, driven by an injection-rotating single cylinder with a screw/barrel inner diameter of 30 mm and an overall length of 520 mm. The maximum shot capacity was calculated as the theoretical shot volume multiplied by the PS plastic coefficient of 0.91, and the maximum setting value for injection pressure was 170 bar. The selection of these four polymers, combined with the controlled molding system, therefore provides a robust basis for comprehensively evaluating the influence of draft angle on both sur-face texture replication and dimensional accuracy, while simultaneously addressing the contrasting behaviors of commodity and engineering plastics.

### 2.3. Measurement System and Related Information

Surface roughness was assessed using 20 specimens produced under identical molding conditions. The influence of polymer type—polypropylene (PP), polyoxymethylene (POM), acrylonitrile-butadiene-styrene (ABS), and polycarbonate (PC)—on roughness and its variability after molding, together with the associated effects on dimensional accuracy, was systematically investigated. Eight draft angles (0°, 0.5°, 1°, 1.5°, 2°, 2.5°, 3°, and 3.5°) were examined to capture their influence on surface replication. The complete molding parameters and material-specific settings for PP, POM, ABS, and PC are summarized in Table 1.

**Figure 4:**
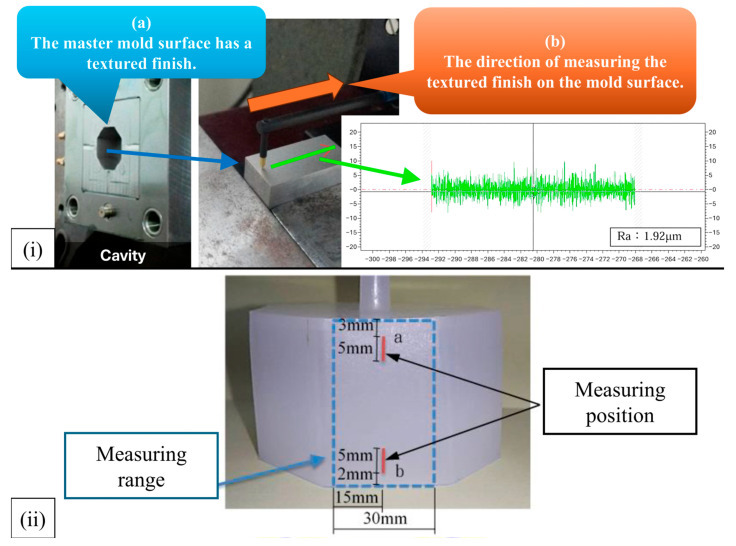
Schematic of the surface roughness measurement of the finished product after molding: (**i**) mold surface measurement; (**ii**) specimen surface measurement.

Differences were observed in plasticizing and injection times among the four polymers, reflecting their distinct rheological and thermal behaviors. Semi-crystalline polymers exhibited longer plasticizing and injection durations (POM: 10 s and 1.25 s; PP: 7 s and 0.75 s) compared with amorphous polymers (PC: 12 s and 1.0 s; ABS: 7 s and 0.72 s). These variations indicate that crystalline materials require longer melt preparation and filling stages due to higher melting temperatures and crystallization kinetics, while amorphous polymers, despite shorter injection times, show prolonged plasticizing when higher processing temperatures are needed. Such differences in melt dynamics are expected to influence surface texture replication and dimensional stability across the investigated draft angles. In addition, to accommodate the differences among material types—including commodity and engineering plastics as well as crystalline and amorphous polymers, the melt temperature was set to 200 °C for commodity plastics and 250 °C for engineering plastics, while the mold temperature was set to 50 °C for crystalline and 70 °C for amorphous materials. The machine parameters were selected based on process stability and cross-material comparability. The screw speed (100 rpm) and back pressure (5 bar) ensured homogeneous melt plasticization without excessive shear heating. The material usage (32 mm shot size) corresponded to approximately 100% cavity filling to maintain consistent packing density. The injection speed (70 mm/s) and packing pressure (20%) were chosen to guarantee complete filling for polymers of different viscosities while preventing flash formation. The packing pressure switching point (10 mm) secured a smooth transition between filling and packing. Both packing time and cooling time were fixed at 10 s, which was verified through short-shot trials to provide sufficient solidification and dimensional stability. These parameters were determined according to the standard stable operating range of a 60-ton injection molding machine (CLF-60TX) and the recommendations from the Moldex3D material database. They were validated experimentally and then held constant for all materials to eliminate process-related variations, ensuring that the observed differences primarily reflected the effects of draft angle and polymer characteristics. Figure 4 illustrates the measurement method of surface roughness in both the mold and the molded specimens.

**Figure 5:**
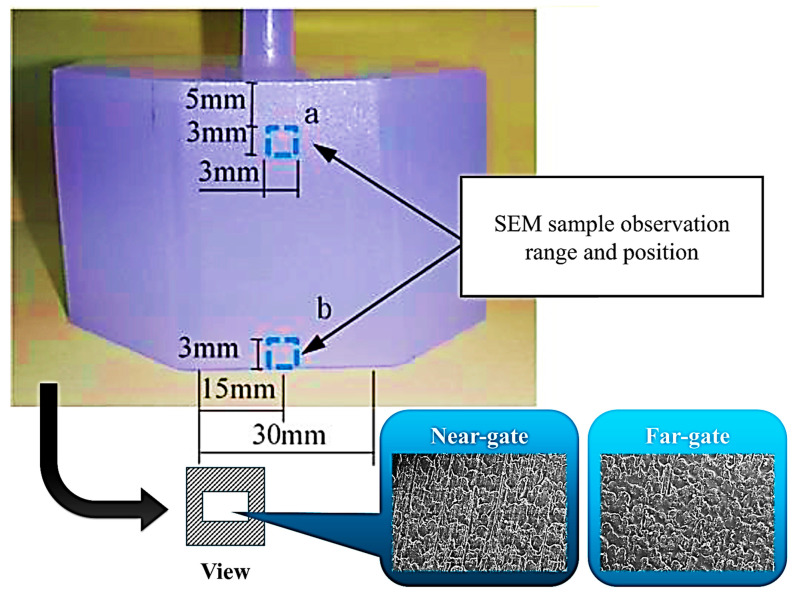
Schematic of scanning electron microscopy observation position of surface texture in the specimens.

In Figure 4i, the master mold cavity surface was fabricated with a textured finish, and its reference roughness was characterized prior to molding using a profilometer along the indicated direction, yielding a baseline roughness value of approximately Ra of 1.92 μm. This ensured that the mold surface quality was well defined before evaluating replication on the molded parts. In Figure 4ii, the measuring range and positions on the specimen surface are indicated. Roughness was measured at two specific locations: (a) near the top edge (5 mm below the sprue) and (b) near the bottom edge (5 mm from the base), with a vertical measuring span of 30 mm. On the other hand, Figure 5 shows the regions selected for scanning electron microscopy (SEM) observation of the replicated surface textures. Two specific areas were analyzed: (a) the near-gate region, located approximately 5 mm below the gate, and (b) the far-gate region, located near the bottom edge, 15 mm from the gate. Each observation area covered approximately 3 mm × 3 mm to ensure representative sampling of the surface morphology.

**Figure 6:**
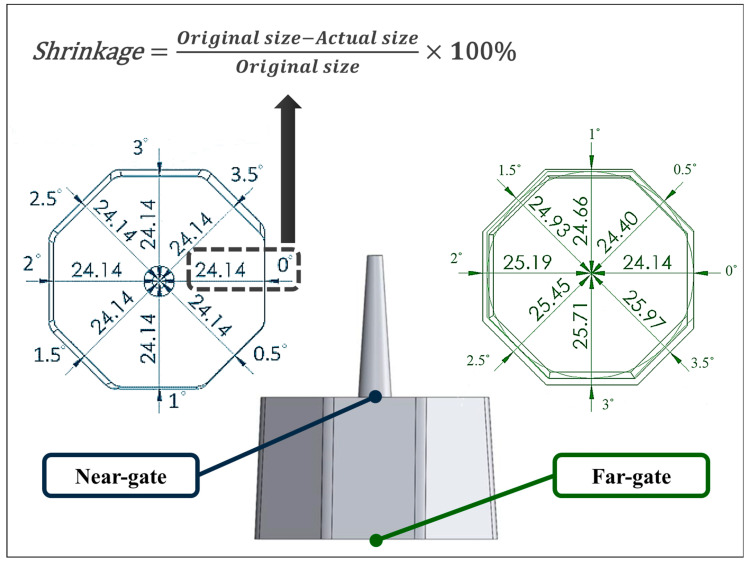
Schematic diagram of the measurement of the finished product after shrinkage and the formula for its calculation.

Figure 6 illustrates the schematic of dimensional measurement used for shrinkage evaluation. The left-hand side depicts the original geometric design of the specimen and sprue. Dimensional measurements were taken radially outward from the center of the part, where the origin was defined at (X: 0, Y: 0). For instance, the distance from the center to the 0° draft angle position (X: 24.14, Y: 0) was recorded. Measurements were conducted at both the near-gate (adjacent to the sprue) and far-gate (end of flow) regions, totaling sixteen reference points. After molding and cooling, the actual specimen widths at the near-gate and far-gate positions were measured, and the deviations from the designed cavity dimensions were calculated to determine linear shrinkage. This measurement strategy established a direct correlation between draft angle and polymer type with respect to dimensional accuracy. The octagonal geometry facilitated consistent evaluation across all draft angles (0–3.5°), while comparison between near-gate and far-gate regions revealed the influence of local flow and cooling gradients on shrinkage behavior. To ensure the accuracy and representativeness of the measurements, five specimens are randomly selected for measurement, and the averages are calculated along with their standard errors. The detailed outcomes are presented in Table 2. Given the uncomplicated geometry of the final specimen and the precise measurement technique employed, the likelihood of errors is minimal.

## 3. Results

**Figure 7:**
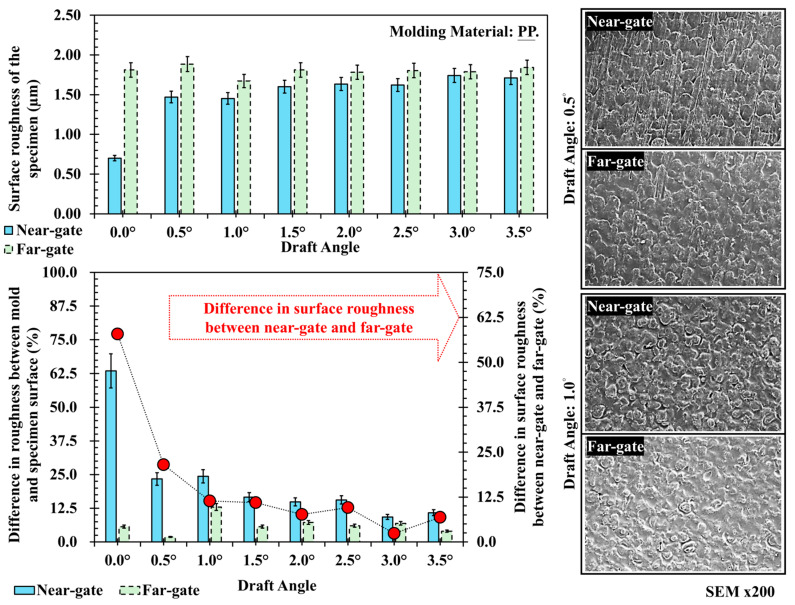
Trend chart and scanning electron microscope image of surface roughness at different draft angles and measurement positions for PP, and its difference in surface roughness between near-gate and far-gate (dotted-line).

Figure 7 presents the trend of surface roughness and corresponding SEM observations of polypropylene (PP) at different draft angles. The data show that the far-gate region consistently exhibits higher roughness than the near-gate, with values maintained between 1.6 and 1.8 μm and only slight variation. In contrast, the near-gate roughness is the lowest at a 0° draft angle, approximately 0.7 μm, and gradually increases with draft angle before stabilizing. This phenomenon can be interpreted from the perspective of melt flow behavior. As the molten PP advances through the cavity, the pressure and shear rate decrease progressively along the flow path. In the far-gate region, the reduced flow velocity and pressure result in slower surface wetting and insufficient replication, leading to rougher surface textures. Conversely, at the near-gate, the higher shear rate promotes better melt penetration into micro-asperities of the mold surface, generating smoother textures at lower draft angles. However, as the draft angle increases, the contact area between the molded surface and the cavity wall decreases, thereby reducing friction and ejection resistance, which helps preserve the surface features and improves texture retention near the gate. SEM images confirm this interpretation: at low draft angles of 0.5° and 1.0°, the near-gate shows evident flow marks and directional alignment due to strong shear flow along the cavity wall, while the far-gate exhibits more uniform and deeper texture patterns formed under slower flow and higher-pressure decay.

**Figure 8:**
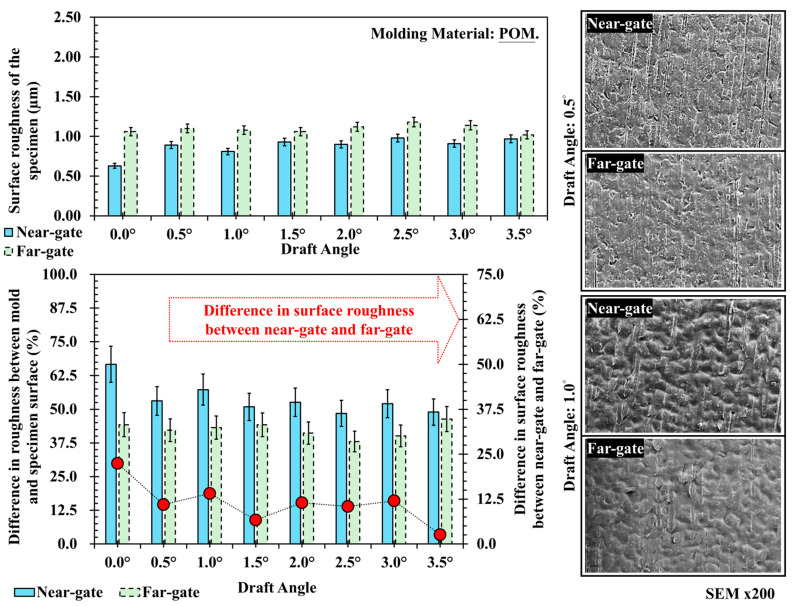
Trend chart and scanning electron microscope image of surface roughness at different draft angles and measurement positions for POM, and its difference in surface roughness between near-gate and far-gate (dotted-line).

Figure 8 shows the surface roughness and corresponding SEM features of polyoxymethylene (POM) at different draft angles. The roughness values are distributed between 0.7 and 1.1 μm, with the far-gate consistently higher than the near-gate. As the draft angle increases, the difference between the two gradually decreases, particularly beyond 3.0°, where the values converge. This behavior can be interpreted from the viewpoint of melt flow and pressure distribution. As the molten POM flows through the cavity, the pressure and shear stress decrease toward the flow end, resulting in weaker cavity-wall contact and slightly rougher textures at the far-gate. At smaller draft angles, the near-gate region experiences greater ejection resistance and uneven cooling, which can cause minor surface deformation or texture loss. Increasing the draft angle reduces friction during ejection and stabilizes melt flow near the wall, enabling better texture preservation at the near-gate and thus narrowing the roughness difference. SEM images corroborate this trend: at 0.5°, the near-gate surface shows string-like flow marks and irregular texture alignment, while at 1.0°, surface replication becomes more uniform, with deeper and more consistent texture profiles.

**Figure 9:**
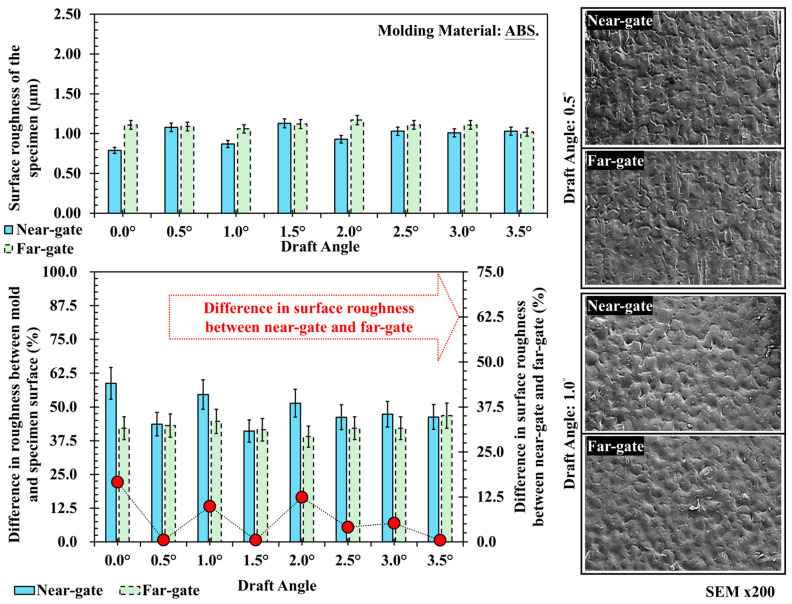
Trend chart and scanning electron microscope image of surface roughness at different draft angles and measurement positions for ABS, and its difference in surface roughness between near-gate and far-gate (dotted-line).

**Figure 10:**
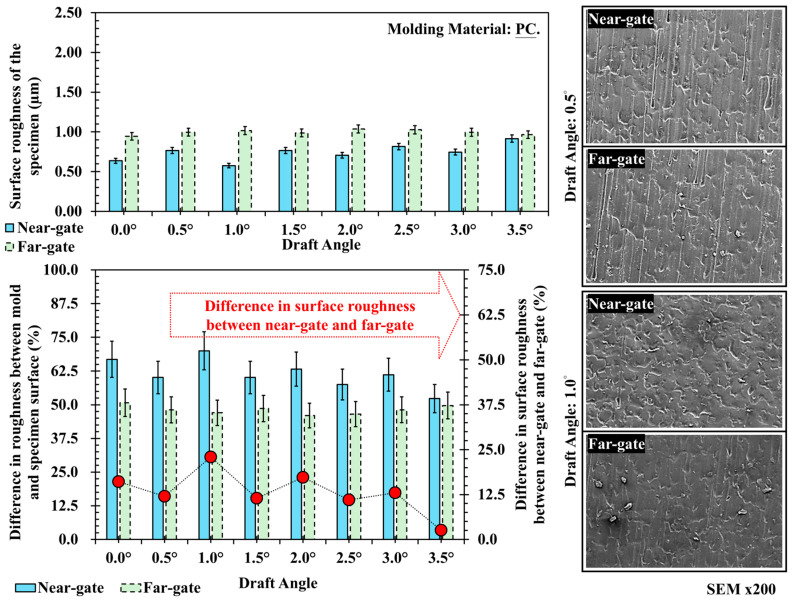
Trend chart and scanning electron microscope image of surface roughness at different draft angles and measurement positions for PC, and its difference in surface roughness between near-gate and far-gate (dotted-line).

Figure 9 presents the surface roughness and SEM results for acrylonitrile–butadiene–styrene (ABS). The roughness values range from 0.8 to 1.1 μm, showing minimal difference between near- and far-gate regions and only slight variation across draft angles. This can be explained by the flow stability of amorphous ABS. During filling, its relatively uniform viscosity and slower cooling rate promote steady flow and homogeneous pressure distribution, resulting in consistent surface replication regardless of draft angle. Since the molecular structure of ABS remains disordered during cooling, texture fidelity primarily depends on the mold surface precision and melt filling pressure rather than draft angle geometry. SEM observations confirm that both near- and far-gate surfaces exhibit fine and uniform textures with only minor differences in depth, where the far-gate displays slightly more pronounced features due to reduced local shear during filling. Figure 10 illustrates the surface roughness and SEM images of polycarbonate (PC) at different draft angles. The roughness values range from 0.7 to 1.0 μm, with the far-gate slightly higher than the near-gate, and only minor fluctuations observed with draft angle variation. As an engineering plastic processed at high temperature and moderate viscosity, PC exhibits highly stable flow and low shrinkage, so draft angle has a limited effect on its surface replication. However, at smaller draft angles, the higher demolding stress and uneven cooling at the part–mold interface may cause local scratches or subtle texture distortion. SEM images verify these findings: at 0.5° and 1.0°, both near- and far-gate regions show uniform and clearly defined textures, with only a few small defects caused by localized stress concentration during demolding.

**Figure 11:**
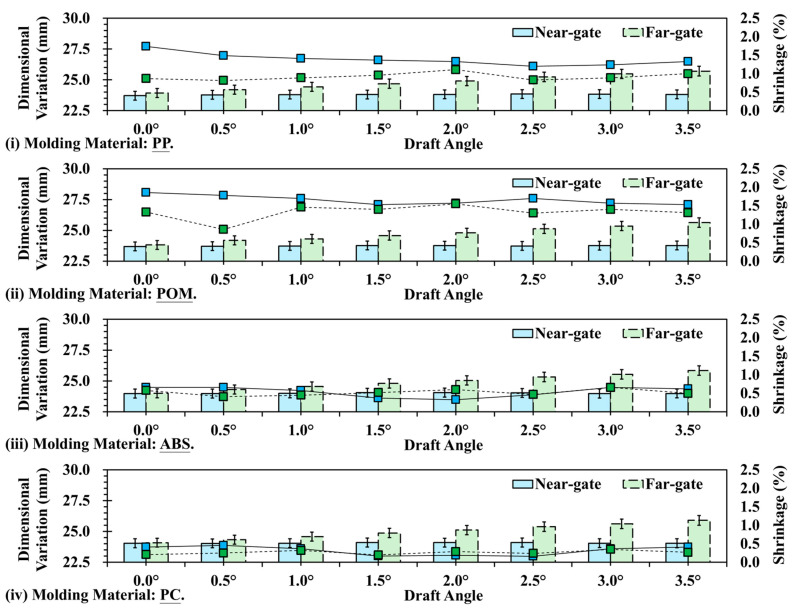
Trend chart of shrinkage variation at different draft angle and measurement positions: (**i**) PP; (**ii**) POM; (**iii**) ABS; (**iv**) PC.

Figure 11 shows the shrinkage variation trends of four materials (PP, POM, ABS, and PC) under different draft angles, comparing the near-gate and far-gate positions. From the results, it can be observed that crystalline materials (PP and POM) exhibit significantly higher shrinkage than amorphous materials (ABS and PC), and the draft angle has a greater influence on the shrinkage difference between near- and far-gate regions. In contrast, the shrinkage variation of amorphous materials remains relatively small, with limited influence from draft angle, thereby demonstrating better dimensional stability. This finding is consistent with the earlier analysis of surface roughness and texture replication, highlighting the dominant role of crystallinity in determining shrinkage behavior and sensitivity to draft angle design. For PP, the shrinkage at the near-gate is clearly higher than that at the far-gate, reaching up to 0.45 mm at a 0° draft angle. As the draft angle increases, the near-gate shrinkage gradually decreases and stabilizes around 2.5° (Figure 11i). The far-gate shrinkage remains relatively constant at approximately 0.2 mm, with only minor variation. This result indicates that PP, as a semi-crystalline polymer, undergoes higher shrinkage during cooling due to crystalline structure formation, while increasing the draft angle can mitigate mold-wall friction and stress concentration in the near-gate region, thereby reducing localized shrinkage. Similarly, Figure 11ii shows that POM exhibits a smaller difference in shrinkage between near- and far-gate regions compared with PP, although the trend of near-gate > far-gate persists. At a 0° draft angle, the near-gate shrinkage is about 0.45 mm, while the far-gate shrinkage is about 0.35 mm. With increasing draft angle, the values gradually converge. This behavior corresponds to the high crystallinity of POM, which results in a high but more uniform shrinkage rate. Proper draft angle design can further reduce demolding friction, allowing the near-gate shrinkage to become closer to that of the far-gate. In addition, the characteristics of amorphous materials reveal that ABS and PC exhibit overall shrinkage values significantly lower than PP and POM, with minimal differences between near- and far-gate regions. Their shrinkage values remain within the range of 0.05–0.15 mm, showing only slight fluctuations with draft angle (Figure 11 iii,iv). This can be attributed to the amorphous nature of these polymers, in which the molecular chains are arranged in a disordered manner during cooling, resulting in extremely low shrinkage and excellent dimensional stability. Therefore, the draft angle has only a limited effect on the shrinkage behavior of amorphous materials.

**Figure 12:**
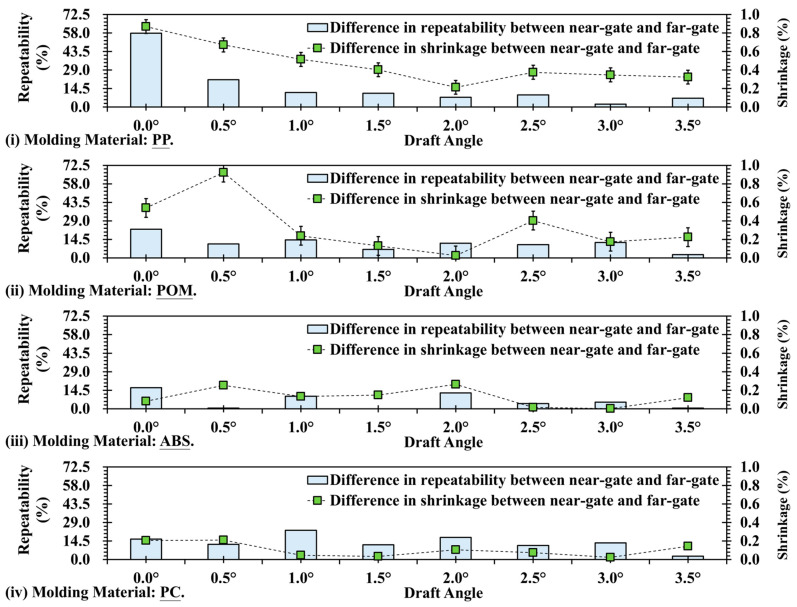
Trend chart of difference in replication (solid line) and shrinkage (dotted line) at different draft angle and measurement positions: (**i**) PP; (**ii**) POM; (**iii**) ABS; (**iv**) PC.

Figure 12 shows a comparison of surface texture replication differences and shrinkage differences between the near-gate and far-gate regions of four materials under various draft angles. This comparison enables the simultaneous observation of how draft angle influences the consistency of surface texture and dimensional deformation, while also reflecting the differences between crystalline and amorphous materials. For PP, at a 0° draft angle, the replication difference exceeds 50%, and the shrinkage difference is also at a high level, indicating significant disparities between the near- and far-gate regions. As the draft angle increases, both values decrease and stabilize beyond 2°, maintaining differences below 10%. This corresponds to the semi-crystalline nature of PP, where increasing draft angle reduces demolding friction, leading to improved consistency in surface texture and dimensional shrinkage (Figure 12i). For POM, the initial replication and shrinkage differences are lower than those of PP but exhibit a similar trend. At 0°, the difference is about 25%, which gradually decreases with increasing draft angle and reaches a minimum at 1.5–2.0° (Figure 12ii). This indicates that although POM is a highly crystalline material, its shrinkage is more uniform, and an appropriate draft angle further reduces near- and far-gate discrepancies, thereby improving consistency. In contrast, ABS exhibits generally low difference values, with a notable peak of over 35% appearing around 2.0°, while at most other draft angles the differences remain within 10% (Figure 12iii). This reflects the amorphous nature of ABS, which undergoes low and stable cooling shrinkage. Its texture replication primarily depends on mold surface precision and filling pressure rather than draft angle. However, localized variations may still occur due to subtle differences in flow balance, gate pressure, or demolding friction at specific regions of the cavity. These factors can locally alter melt velocity, surface wetting, or cooling uniformity, leading to slight deviations in texture depth or gloss. Furthermore, PC shows fluctuating curves for both replication and shrinkage differences, ranging between 10% and 30%, without a significant decline as the draft angle increases (Figure 12iv). This suggests that PC, as an amorphous engineering plastic, possesses excellent dimensional stability, yet its higher molding temperature may intensify local frictional heating or uneven cooling near ejector pins and cavity corners, resulting in minor surface inconsistencies. Consequently, the influence of draft angles on PC remains relatively limited compared with that of localized flow and thermal effects.

## 4. Discussion

This study systematically evaluated how draft angle design influences surface texture replication and dimensional stability at near-gate and far-gate locations across four thermoplastics. The results indicate that draft angle mainly affects demolding friction and localized shrinkage, thereby improving texture clarity and positional consistency, while polymer crystallinity governs the overall levels of surface roughness and shrinkage and determines process sensitivity to draft angle. Notable research results are as follows:

- Draft angle strongly improves texture clarity but has a limited impact on global deformation trends. Increasing draft angle enhances texture retention at the near-gate location while only gradually moderating overall shrinkage. For PP, near-gate roughness rises from about 0.7 μm at 0° and stabilizes with angle, whereas far-gate roughness remains around 1.6 to 1.8 μm.
- Crystalline polymers exhibit higher roughness and shrinkage than amorphous polymers. Roughness is as follows: PP roughness is greater than POM and that of ABS is greater than PC. Shrinkage is more pronounced in crystalline materials with an overall order of POM, PP, ABS, and PC, consistent with crystallization-induced local shrinkage and higher ejection friction.
- Draft angle effectively narrows near-gate and far-gate discrepancies, especially for crystalline polymers. In PP and POM, increasing draft angle markedly reduces differences in roughness and shrinkage. For PP, replication difference exceeds 50 percent at 0° and drops below 10 percent beyond 2°. For POM, the initial difference of about 25 percent reaches a minimum around 1.5° to 2.0°.
- Amorphous polymers are less sensitive to draft angles and maintain low roughness with high dimensional stability. ABS and PC show roughness ranges of about 0.8 to 1.1 μm and 0.7 to 1.0 μm, respectively, and shrinkage of about 0.05 to 0.15 mm with small variations across angles. ABS occasionally exhibits a peak replication difference above 35 percent near 2.0°, likely due to localized flow or friction. PC fluctuates between about 10 and 30 percent, reflecting high-temperature molding with local cooling and friction effects.
- Larger draft angles should be used for crystalline polymers, and moderate angles should be used for amorphous polymers while prioritizing mold finish and filling control. Practical ranges are about 1.5° to 2.5° for PP and POM to lower demolding friction and improve near–far consistency. For ABS and PC, about 0.5° to 1.5° is generally sufficient, with emphasis on mold surface quality and stable filling pressure to avoid local defects.

## Figures and Tables

**Table 1:** Basic parameter setting for the manufacturing of the injection-molded different molding materials.

Parameter	PP	POM	ABS	PC
Barrel heating-film temperature (melt temperature)	200 °C	250 °C	200 °C	250 °C
Mold heating, water temperature (mold temperature)	50 °C	70 °C	50 °C	70 °C
Plasticizing time	7 s	10 s	7 s	12 s
Injection time	0.75 s	1.25 s	0.72 s	1 s
Screw speed	100 rpm	100 rpm	100 rpm	100 rpm
Back pressure	5 bar	5 bar	5 bar	5 bar
Material usage	32 mm	32 mm	32 mm	32 mm
Injection pressure	100%	100%	100%	100%
Injection speed	70 mm/s	70 mm/s	70 mm/s	70 mm/s
Packing pressure switching point	10 mm	10 mm	10 mm	10 mm
Packing pressure	20%	20%	20%	20%
Packing time	10 s	10 s	10 s	10 s
Cooling time	10 s	10 s	10 s	10 s

**Table 2:** The experimental measurement of the roughness and dimensions of the individual of the Injection-Molded specimens.

Material: PP.	Surface Roughness (µm)				Dimensional Variation (mm)				Shrinkage (%)			
	Near-Gate		Far-Gate		Near-Gate		Far-Gate		Near-Gate		Far-Gate	
EXP.	AVG.	ANOVA	AVG.	ANOVA	AVG.	ANOVA	AVG.	ANOVA	AVG.	ANOVA	AVG.	ANOVA
0.0	0.70	0.0019%	1.81	0.0020%	23.72	0.15034%	23.93	0.08603%	1.74	0.06579%	0.87	0.01827%
0.5	1.47	0.0026%	1.88	0.0001%	23.78	0.15110%	24.20	0.08798%	1.49	0.04824%	0.82	0.01623%
1.0	1.45	0.0036%	1.67	0.0019%	23.80	0.15135%	24.44	0.08973%	1.41	0.04320%	0.89	0.01912%
1.5	1.60	0.0020%	1.81	0.0020%	23.81	0.15148%	24.69	0.09158%	1.37	0.04078%	0.96	0.02224%
2.0	1.63	0.0001%	1.78	0.0012%	23.82	0.15161%	24.91	0.09322%	1.33	0.03844%	1.11	0.02974%
2.5	1.62	0.0023%	1.80	0.0068%	23.85	0.15199%	25.24	0.09570%	1.20	0.03129%	0.83	0.01663%
3.0	1.74	0.0009%	1.79	0.0014%	23.84	0.15186%	25.48	0.09753%	1.24	0.03341%	0.89	0.01912%
3.5	1.71	0.0012%	1.84	0.0018%	23.82	0.15161%	25.71	0.09930%	1.33	0.03844%	1.00	0.02413%
Material: POM.	Surface roughness (µm)				Dimensional variation (mm)				Shrinkage (%)			
	Near-gate		Far-gate		Near-gate		Far-gate		Near-gate		Far-gate	
EXP.	AVG.	ANOVA	AVG.	ANOVA	AVG.	ANOVA	AVG.	ANOVA	AVG.	ANOVA	AVG.	ANOVA
0.0	0.64	0.0621%	1.07	0.0617%	23.69	0.14996%	23.82	0.13693%	1.86	0.05197%	1.33	0.04269%
0.5	0.90	0.0562%	1.11	0.0584%	23.71	0.15021%	24.19	0.14122%	1.78	0.04760%	0.86	0.01785%
1.0	0.82	0.0617%	1.09	0.0562%	23.73	0.15046%	24.30	0.14251%	1.70	0.04342%	1.46	0.05144%
1.5	0.94	0.0725%	1.07	0.0562%	23.77	0.15097%	24.58	0.14581%	1.53	0.03517%	1.40	0.04730%
2.0	0.91	0.0617%	1.13	0.0617%	23.76	0.15084%	24.80	0.14843%	1.57	0.03703%	1.55	0.05798%
2.5	0.99	0.0617%	1.19	0.0617%	23.73	0.15046%	25.12	0.15229%	1.70	0.04342%	1.30	0.04079%
3.0	0.92	0.0617%	1.15	0.0617%	23.76	0.15084%	25.35	0.15509%	1.57	0.03703%	1.40	0.04730%
3.5	0.98	0.0617%	1.03	0.0617%	23.77	0.15097%	25.63	0.15853%	1.53	0.03517%	1.31	0.04142%
Material: ABS.	Surface roughness (µm)				Dimensional variation (mm)				Shrinkage (%)			
	Near-gate		Far-gate		Near-gate		Far-gate		Near-gate		Far-gate	
EXP.	AVG.	ANOVA	AVG.	ANOVA	AVG.	ANOVA	AVG.	ANOVA	AVG.	ANOVA	AVG.	ANOVA
0.0	0.79	0.0566%	1.11	0.0582%	23.98	0.15365%	24.00	0.01051%	0.66	0.05197%	0.58	0.00812%
0.5	1.08	0.0566%	1.09	0.0573%	23.98	0.15365%	24.30	0.01051%	0.66	0.04760%	0.41	0.00406%
1.0	0.87	0.0573%	1.06	0.0566%	24.00	0.15391%	24.55	0.00812%	0.58	0.04342%	0.45	0.00489%
1.5	1.13	0.0582%	1.12	0.0566%	24.05	0.15455%	24.80	0.00330%	0.37	0.03517%	0.52	0.00653%
2.0	0.93	0.0595%	1.17	0.0582%	24.06	0.15468%	25.04	0.00263%	0.33	0.03703%	0.60	0.00869%
2.5	1.03	0.0595%	1.11	0.0582%	24.03	0.15429%	25.33	0.00511%	0.46	0.04342%	0.47	0.00533%
3.0	1.01	0.0582%	1.11	0.0566%	23.98	0.15365%	25.54	0.01051%	0.66	0.03703%	0.66	0.01051%
3.5	1.03	0.0582%	1.02	0.0566%	23.99	0.15378%	25.84	0.00928%	0.62	0.03517%	0.50	0.00603%
Material: PC.	Surface roughness (µm)				Dimensional variation (mm)				Shrinkage (%)			
	Near-gate		Far-gate		Near-gate		Far-gate		Near-gate		Far-gate	
EXP.	AVG.	ANOVA	AVG.	ANOVA	AVG.	ANOVA	AVG.	ANOVA	AVG.	ANOVA	AVG.	ANOVA
0.0	0.64	0.0855%	0.95	0.0828%	24.04	0.15442%	24.09	0.15506%	0.41	0.00449%	0.21	0.00118%
0.5	0.77	0.0828%	1.00	0.0855%	24.03	0.15429%	24.34	0.15830%	0.46	0.00565%	0.25	0.00167%
1.0	0.58	0.0828%	1.02	0.0882%	24.05	0.15455%	24.58	0.16144%	0.37	0.00366%	0.32	0.00274%
1.5	0.77	0.0828%	0.99	0.0868%	24.10	0.15519%	24.88	0.16540%	0.17	0.00077%	0.20	0.00107%
2.0	0.71	0.0828%	1.04	0.0882%	24.10	0.15519%	25.12	0.16861%	0.19	0.00096%	0.29	0.00225%
2.5	0.82	0.0828%	1.03	0.0882%	24.10	0.15519%	25.39	0.17225%	0.16	0.00068%	0.24	0.00154%
3.0	0.75	0.0828%	1.00	0.0830%	24.05	0.15455%	25.62	0.17539%	0.37	0.00366%	0.35	0.00327%
3.5	0.92	0.0828%	0.97	0.0830%	24.04	0.15442%	25.90	0.17924%	0.41	0.00449%	0.27	0.00195%

## Data Availability

The original contributions presented in this study are included in the article. Further inquiries can be directed to the corresponding author.
